# Crystal structure of aqua-1κ*O*-{μ-2-[(2-hydroxy­ethyl)methylamino]ethanolato-2:1κ^4^ *O* ^1^,*N*,*O* ^2^:*O* ^1^}[μ-2,2′-(methylimino)diethanolato-1:2κ^4^ *O*,*N*,*O*′:*O*]dithiocyanato-1κ*N*,2κ*N*-chromium(III)copper(II)

**DOI:** 10.1107/S2056989015015601

**Published:** 2015-08-26

**Authors:** Julia A. Rusanova, Valentina V. Semenaka, Viktoriya V. Dyakonenko, Oleg V. Shishkin

**Affiliations:** a Department of Chemistry, Taras Shevchenko National University, 64/13, Volodymyrska Street, Kyiv, 01601, Ukraine; b Institute for Scintillation Materials, "Institute for Single Crystals", National Academy of Sciences of Ukraine, 60 Lenina Avenue, Kharkiv 61001, Ukraine

**Keywords:** crystal structure, *N*-methyldi­ethano­lamine, heterometal Cu^II^/Cr^III^ complex

## Abstract

The title compound, [CrCu(C_5_H_11_NO_2_)(C_5_H_12_NO_2_)(NCS)_2_(H_2_O)] or [Cr(μ-mdea)Cu(μ-Hmdea)(NCS)_2_H_2_O], (where mdeaH_2_ is *N*-methylethanolamine, C_5_H_13_NO_2_) is formed as a neutral heterometal Cu^II^/Cr^III^ complex. The mol­ecular structure of the complex is based on a binuclear {CuCr(μ-O)_2_} core. The coordination environment of each metal atom involves the *N*,*O*,*O* atoms of the tridentate ligand, one bridging O atom of the ligand and the N atom of the thio­cyanato ligands. The Cu^II^ ion adopts a distorted square-pyramidal coordination while the Cr^III^ ion has a distorted octa­hedral coordination geometry completed by the aqua ligand. In the crystal, the binuclear complexes are linked *via* two pairs of O—H⋯O hydrogen bonds to form inversion dimers, which are arranged in columns parallel to the *a* axis. In the μ-mdea ligand two –CH_2_ groups and the methyl group were refined as disordered over two sets of sites with equal occupancies. The structure was refined as a two-component twin with a twin scale factor of 0.242 (1).

## Abstract 2

[Cr(μ-mdea)Cu(μ-Hmdea)(NCS)_2_H_2_O], (where *mdeaH_2_* is *N*-methyldi­ethano­lamine) is formed as a neutral heterometal Cu^II^/Cr^III^ complex whose mol­ecular structure is based on a binuclear {CuCr(μ-O)_2_} core. In the crystal, the binuclear complexes are linked *via* two pairs of O—H⋯O hydrogen bonds to form inversion dimers, which are arranged in columns parallel to the *a* axis.

## Chemical context   

The search for heterometallic complexes has been stimulated by the general inter­est in combining different metal atoms within one assembly, since even the synthesis of such complexes often represents a non-trivial task. In addition, it was found that such compounds are potential novel magnetic materials (Gheorghe *et al.*, 2010 ▸; Long *et al.*, 2010 ▸; Visinescu *et al.*, 2009 ▸; Amiri *et al.*, 2010 ▸; Timco *et al.*, 2008 ▸). Polydentate alkoxido ligands possessing versatile bridging modes were recognized as promising reagents for the synthesis of new heterometallic complexes. In particular di­ethano­lamine and its *N*-alkyl derivatives are recognized *N*,*O* ligands that possess an inter­esting coordination chemistry and are thus often used for the design of various multimetallic cores and polymeric assemblies (Allen, 2002 ▸; Singh & Mehrotra, 2004 ▸; Verkade, 1993 ▸; Stamatatos *et al.*, 2008 ▸; Beedle *et al.*, 2008 ▸; Kirillov *et al.*, 2008 ▸). Great inter­est in the synthesis and investigation of polynuclear chromium containing compounds dates from the late 90s, mostly due to the works of Winpenny and co-workers devoted to magnetic studies of high-nuclear cages and wheels (McInnes *et al.*, 2005 ▸; Affronte *et al.*, 2005 ▸). As has been shown in our previous publications, the synthetic approach named ‘direct synthesis of coordination compounds’ [Pryma *et al.*, 2003 ▸; Nesterov *et al.*, 2011 ▸, 2012 ▸; Nesterova (Pryma) *et al.*, 2004 ▸; Nesterova *et al.* 2005 ▸; Buvaylo *et al.*, 2005 ▸] is an efficient method to obtain novel heterobi- (Buvaylo *et al.*, 2005 ▸), heterotrimetallic (Nesterov *et al.*, 2011 ▸), polymeric [Nesterova (Pryma) *et al.*, 2004 ▸; Nesterova *et al.*, 2005 ▸, 2008 ▸] and polynuclear (Nesterov *et al.*, 2012 ▸) complexes. In a continuation of our investigations in the field of the ammonium salt route for direct synthesis (Pryma *et al.*, 2003 ▸; Nikitina *et al.*, 2008 ▸) the title compound [Cr(μ-mdea)Cu(μ-Hmdea)(NCS)_2_H_2_O] (where mdeaH_2_ is *N*-methylethanolamine) was prepared using copper powder, Reineckes salt, ammonium thio­cyanate and a non-aqueous solution of mdeaH_2_ in air.
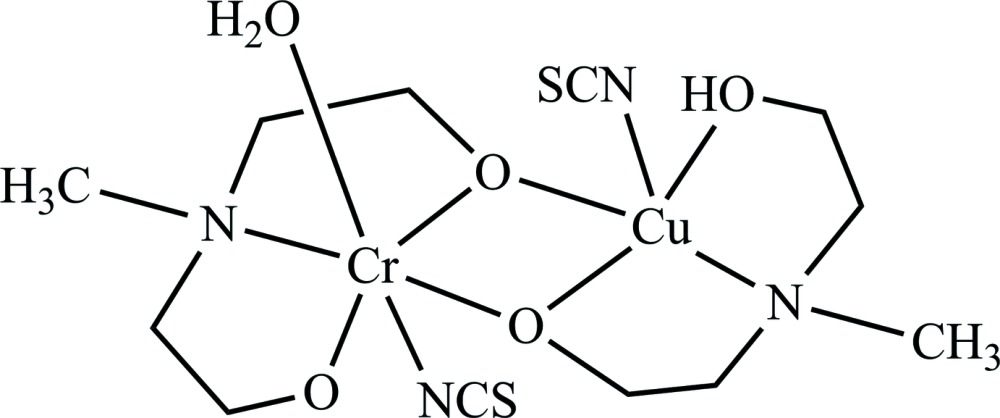


## Structural commentary   

**Figure 1:**
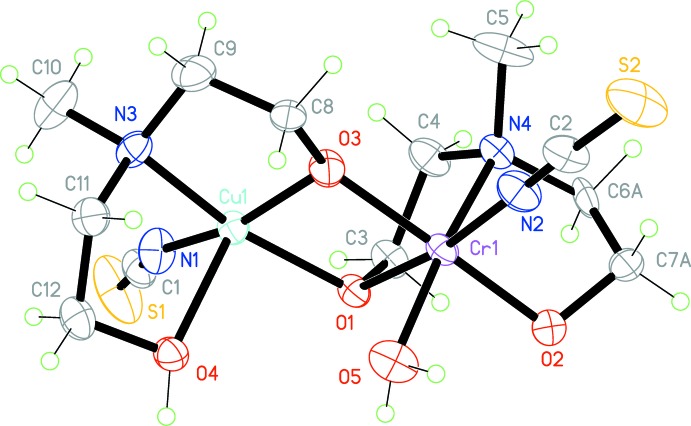
The mol­ecular structure of the title complex with 30% probability displacement ellipsoids

The mol­ecular structure of the title complex (Fig. 1 ▸) is based on a binuclear {CuCr(μ-O)_2_} core. Each ligand (protonated and deprotonated) displays tridentate coordination by N and O atoms to a specific metal atom as well by a bridging O atom to the neighbouring metal atom. Thus the Cu^II^ ion is penta­coordinated by the μ-oxygen (O1, O3) atoms of the proton­ated and deprotonated ligands, the N3 amino nitro­gen atom of the mdea ligand and atom N1 of the ­thio­cyanato ligand in the basal plane, and by the remaining oxygen atom (O4) of the Hmdea ligand in the apical site, and displays a distorted square-pyramidal coordination geometry. The apical oxygen atom is bound through the Cu1—O4 [2.259 (4) Å] bond, which is typically elongated in comparison to those in basal sites, *i.e.* Cu1—O1 [1.994 (3) Å] and Cu1—O3 [1.909 (4) Å]. The coordination environment of the Cr^III^ atom is completed in a distorted octa­hedral geometry by the additional coordination of atom O5 of the water mol­ecule in an axial position *trans* to the N4 amino nitro­gen atom of the ligand. The Cr—(O,N) bond lengths are within the range 1.912 (4)–2.118 (5) Å.

The binding of each *mdea* ligand involves two five-membered *M*–N–C–C–O chelate rings (*M* = Cu, Cr). The angles N3—Cu1—O4 and N3—Cu1—O3 are 82.2 (2) and 84.0 (2)° respectively. The analogous N4—Cr1—O1 and N4—Cr1—O2 angles are 84.2 (2) and 82.9 (2)°, respectively.

The Cu1–O1–Cr1–O3 core is non-planar, and has both atoms O1 and O3 shifted opposite to the direction of apical oxygen O5 atom of the water mol­ecule. In this core, the Cu1⋯Cr1 separation is 2.998 (1) Å. The representative Cu1—O1—Cr1 and Cu1—O3—Cr1 bond angles are 97.8 (1) and 101.5 (2)° respectively, while the O1—Cr1—O3 and O1—Cu1—O3 bond angles are 78.6 (2) and 79.6 (1)°. The dihedral angle between two Cu–O–Cr planes is 18.49 (15)°.

In general, all bonding parameters and the dimensions of the angles in the title complex are in good agreement with those encountered in related amino­alcohol complexes (Figiel *et al.*, 2010 ▸; Kirillov *et al.*, 2008 ▸; Gruenwald *et al.*, 2009 ▸; Vinogradova *et al.*, 2002 ▸).

## Supra­molecular features   

**Figure 2:**
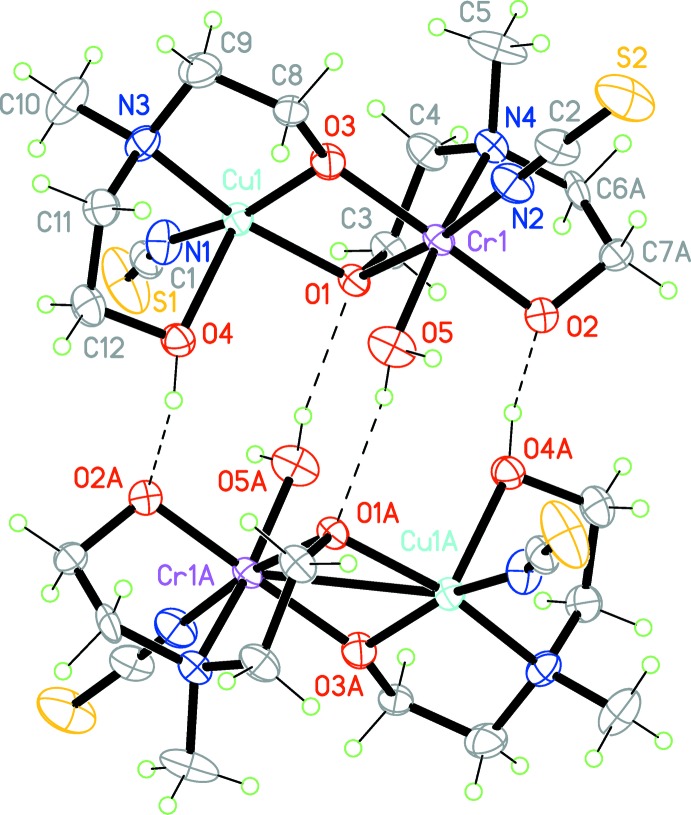
An inversion dimer of title compound connected *via* two pairs of O—H⋯O hydrogen bonds (dashed lines). [Symmetry code: (A) −*x*, −*y*, -*z.*]

**Figure 3:**
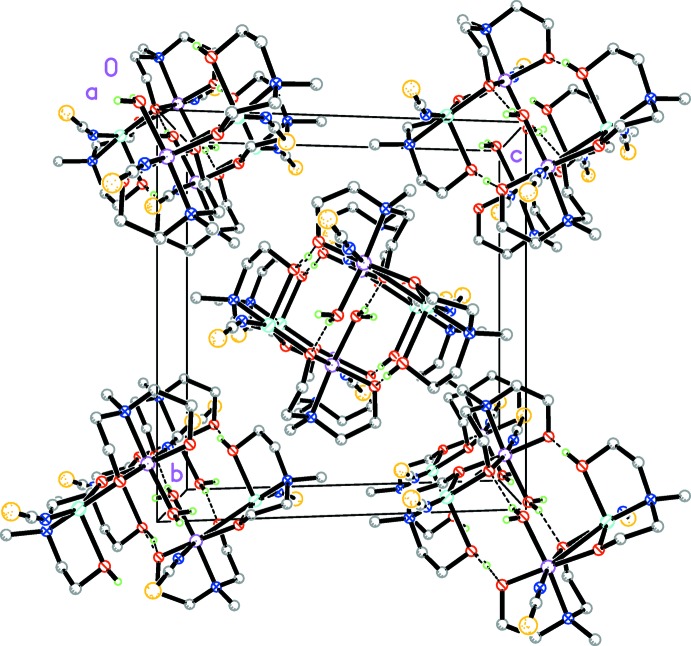
Crystal packing of the title compound viewed along the *a* axis.

In the crystal, the binuclear complexes are linked *via* two pairs of O—H⋯O hydrogen bonds (Table 1 ▸) to form inversion dimers (Fig. 2 ▸), which are arranged in columns parallel to the *a* axis (Fig. 3 ▸).

## Database survey   

A search of the Cambridge Structural Database (Version 5.36; last update February 2015; Groom & Allen, 2014 ▸) for related complexes with *N*-methyldi­ethano­lamine gave 109 hits. Therein closely related structures with a metal–O–metal–O core are heteronuclear complexes with Cu (Figiel *et al.*, 2010 ▸), Ga (Pugh *et al.*, 2012 ▸) and heterometallic with Zn, Co and Cu (Nesterov *et al.*, 2011 ▸).

## Synthesis and crystallization   

Copper powder (0.079 g, 1.25 mmol), NH_4_[Cr(NCS)_4_(NH_3_)_2_]·H_2_O (0.443 g, 1.25 mmol), NH_4_SCN (0.095 g, 1.25 mmol), *N*-methyldi­ethano­lamine (1.3 ml, 1.25 mmol) and methanol (20 ml) were heated in air at 323–333 K and stirred magnetically for 30 min. Deep-blue crystals suitable for crystallographic study were formed by slow evaporation of the resulting solution in air. The crystals were filtered off, washed with dry isopropanol and finally dried *in vacuo* at room temperature. Yield: 0.11 g, 17.7%.

## Refinement   

Crystal data, data collection and structure refinement details are summarized in Table 2 ▸. Hydrogen atoms were located in difference Fourier maps and refined in a riding-model approximation with *U*_iso_ = *nU*_eq_ of the carrier atom (*n* = 1.5 for methyl group and *n* = 1.2 for other hydrogen atoms). Atoms C5, C6 and C7 were refined as disordered over two sets of sites with equal occupancies. The structure was refined as a two-component twin with a twin scale factor of 0.242 (1).

## Supplementary Material

Crystal structure: contains datablock(s) I. DOI: 10.1107/S2056989015015601/lh5774sup1.cif

Structure factors: contains datablock(s) I. DOI: 10.1107/S2056989015015601/lh5774Isup2.hkl

CCDC reference: 1419706

Additional supporting information:  crystallographic information; 3D view; checkCIF report

## Figures and Tables

**Table 1:** Hydrogen-bond geometry (, )

*D*H*A*	*D*H	H*A*	*D* *A*	*D*H*A*
O4H4O2^i^	0.86	1.86	2.595(7)	142
O5H5*B*O1^i^	0.86	2.18	3.014(7)	162

Symmetry code: (i) 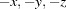.

**Table 2:** Experimental details

Crystal data	
Chemical formula	[Cr(C_5_H_11_NO_2_)Cu(C_5_H_12_NO_2_)(NCS)_2_(H_2_O)]
*M*_r_	485.02
Crystal system, space group	Monoclinic, *P*2_1_/*c*
Temperature (K)	294
*a*, *b*, *c* ()	10.570(3), 14.543(4), 13.940(3)
()	105.571(3)
*V* (^3^)	2064.2(9)
*Z*	4
Radiation type	Mo *K*
(mm^1^)	1.79
Crystal size (mm)	0.50 0.30 0.20

Data collection	
Diffractometer	Agilent Xcalibur, Sapphire3
Absorption correction	Multi-scan (*CrysAlis RED*; Agilent, 2011 ▸)
*T*_min_, *T*_max_	0.829, 1.000
No. of measured, independent and observed [*I* > 2(*I*)] reflections	3596, 3596, 3173
*R*_int_	0.038
(sin /)_max_ (^1^)	0.596

Refinement	
*R*[*F* ^2^ > 2(*F* ^2^)], *wR*(*F* ^2^), *S*	0.057, 0.150, 1.06
No. of reflections	3596
No. of parameters	257
No. of restraints	10
H-atom treatment	H-atom parameters constrained
_max_, _min_ (e ^3^)	0.55, 0.85

Computer programs: *CrysAlis CCD* and *CrysAlis RED* (Agilent, 2011 ▸), *SHELXT* (Sheldrick, 2015*a* ▸), *SHELXL2014*/7 (Sheldrick, 2015*b* ▸), *OLEX2* (Dolomanov *et al.*, 2009 ▸) and *publCIF* (Westrip, 2010 ▸).

## supplementary crystallographic information

### Crystal data

**Table:**

[CrCu(C_5_H_11_NO_2_)(C_5_H_12_NO_2_)(NCS)_2_(H_2_O)]	*F*(000) = 1000
*M**_r_* = 485.02	*D*_x_ = 1.561 Mg m^−^^3^
Monoclinic, *P*2_1_/*c*	Mo *K*α radiation, λ = 0.71073 Å
*a* = 10.570 (3) Å	Cell parameters from 6922 reflections
*b* = 14.543 (4) Å	θ = 3.2–32.9°
*c* = 13.940 (3) Å	µ = 1.79 mm^−^^1^
β = 105.571 (3)°	*T* = 294 K
*V* = 2064.2 (9) Å^3^	Block, blue
*Z* = 4	0.50 × 0.30 × 0.20 mm

### Data collection

**Table:**

Agilent Xcalibur, Sapphire3 diffractometer	3173 reflections with *I* > 2σ(*I*)
ω scans	*R*_int_ = 0.038
Absorption correction: multi-scan (*CrysAlis RED*; Agilent, 2011)	θ_max_ = 25.0°, θ_min_ = 3.3°
*T*_min_ = 0.829, *T*_max_ = 1.000	*h* = −12→12
3596 measured reflections	*k* = −17→17
3596 independent reflections	*l* = −6→16

### Refinement

**Table:**

Refinement on *F*^2^	10 restraints
Least-squares matrix: full	Hydrogen site location: mixed
*R*[*F*^2^ > 2σ(*F*^2^)] = 0.057	H-atom parameters constrained
*wR*(*F*^2^) = 0.150	*w* = 1/[σ^2^(*F*_o_^2^) + (0.0602*P*)^2^ + 5.4768*P*] where *P* = (*F*_o_^2^ + 2*F*_c_^2^)/3
*S* = 1.06	(Δ/σ)_max_ = 0.009
3596 reflections	Δρ_max_ = 0.55 e Å^−^^3^
257 parameters	Δρ_min_ = −0.85 e Å^−^^3^

### Special details

**Table:**

Geometry. All e.s.d.'s (except the e.s.d. in the dihedral angle between two l.s. planes) are estimated using the full covariance matrix. The cell e.s.d.'s are taken into account individually in the estimation of e.s.d.'s in distances, angles and torsion angles; correlations between e.s.d.'s in cell parameters are only used when they are defined by crystal symmetry. An approximate (isotropic) treatment of cell e.s.d.'s is used for estimating e.s.d.'s involving l.s. planes.
Refinement. Refined as a 2-component twin.

### Fractional atomic coordinates and isotropic or equivalent isotropic displacement parameters (Å^2^)

**Table:**

	*x*	*y*	*z*	*U*_iso_*/*U*_eq_	Occ. (<1)
Cu1	0.16459 (6)	0.02434 (4)	0.21704 (5)	0.0350 (2)	
Cr1	0.20912 (8)	0.12817 (5)	0.04319 (6)	0.0337 (2)	
S1	−0.2054 (2)	0.05346 (15)	0.3310 (2)	0.0948 (8)	
S2	0.5965 (2)	0.21482 (17)	−0.04740 (19)	0.0889 (8)	
N1	0.0246 (5)	0.0129 (4)	0.2820 (4)	0.0490 (12)	
N2	0.3744 (5)	0.1577 (4)	0.0039 (4)	0.0525 (13)	
N3	0.3150 (5)	−0.0412 (3)	0.3195 (3)	0.0429 (11)	
N4	0.1924 (5)	0.2607 (3)	0.1015 (3)	0.0421 (11)	
O1	0.0620 (3)	0.0968 (2)	0.1007 (3)	0.0334 (8)	
O2	0.0997 (4)	0.1807 (2)	−0.0761 (3)	0.0450 (9)	
O3	0.3039 (3)	0.0790 (3)	0.1737 (3)	0.0437 (9)	
O4	0.1365 (4)	−0.1172 (2)	0.1477 (3)	0.0428 (9)	
H4	0.0773	−0.1495	0.1068	0.051*	
O5	0.1979 (5)	0.0010 (3)	−0.0255 (4)	0.0679 (13)	
H5A	0.2330	0.0023	−0.0727	0.102*	
H5B	0.1162	−0.0153	−0.0470	0.102*	
C1	−0.0721 (6)	0.0276 (4)	0.3021 (5)	0.0473 (14)	
C2	0.4661 (6)	0.1810 (4)	−0.0151 (5)	0.0486 (14)	
C3	−0.0017 (6)	0.1781 (4)	0.1221 (5)	0.0444 (13)	
H3A	−0.0513	0.1643	0.1696	0.053*	
H3B	−0.0620	0.2012	0.0617	0.053*	
C4	0.1026 (6)	0.2502 (4)	0.1650 (5)	0.0506 (15)	
H4A	0.0606	0.3086	0.1701	0.061*	
H4B	0.1520	0.2319	0.2314	0.061*	
C5A	0.320 (3)	0.307 (3)	0.154 (3)	0.057 (9)	0.5
H5AA	0.3630	0.3283	0.1053	0.085*	0.5
H5AB	0.3759	0.2643	0.1979	0.085*	0.5
H5AC	0.3024	0.3587	0.1914	0.085*	0.5
C6A	0.1142 (18)	0.3163 (13)	0.0171 (12)	0.049 (5)	0.5
H6AA	0.1444	0.3795	0.0237	0.059*	0.5
H6AB	0.0223	0.3155	0.0167	0.059*	0.5
C5B	0.318 (4)	0.289 (3)	0.171 (3)	0.073 (12)	0.5
H5BA	0.3888	0.2752	0.1426	0.110*	0.5
H5BB	0.3301	0.2572	0.2329	0.110*	0.5
H5BC	0.3160	0.3544	0.1826	0.110*	0.5
C6B	0.164 (2)	0.3234 (16)	0.0147 (14)	0.079 (9)	0.5
H6BA	0.2454	0.3415	0.0006	0.095*	0.5
H6BB	0.1211	0.3785	0.0298	0.095*	0.5
C7A	0.1305 (17)	0.2756 (5)	−0.0783 (18)	0.049 (5)	0.5
H7AA	0.0718	0.3052	−0.1354	0.059*	0.5
H7AB	0.2201	0.2835	−0.0823	0.059*	0.5
C7B	0.076 (2)	0.2768 (5)	−0.075 (2)	0.069 (7)	0.5
H7BA	−0.0149	0.2872	−0.0764	0.083*	0.5
H7BB	0.0905	0.3041	−0.1351	0.083*	0.5
C8	0.4311 (5)	0.0428 (5)	0.2170 (5)	0.0512 (16)	
H8A	0.4976	0.0891	0.2184	0.061*	
H8B	0.4477	−0.0096	0.1791	0.061*	
C9	0.4334 (6)	0.0140 (5)	0.3215 (5)	0.0563 (17)	
H9A	0.5116	−0.0221	0.3501	0.068*	
H9B	0.4356	0.0681	0.3627	0.068*	
C10	0.2951 (8)	−0.0468 (5)	0.4206 (4)	0.067 (2)	
H10A	0.2880	0.0142	0.4451	0.100*	
H10B	0.3684	−0.0778	0.4643	0.100*	
H10C	0.2159	−0.0803	0.4177	0.100*	
C11	0.3280 (5)	−0.1354 (4)	0.2823 (4)	0.0468 (14)	
H11A	0.3705	−0.1746	0.3380	0.056*	
H11B	0.3834	−0.1335	0.2370	0.056*	
C12	0.1970 (5)	−0.1760 (4)	0.2294 (5)	0.0529 (15)	
H12A	0.2087	−0.2371	0.2053	0.063*	
H12B	0.1419	−0.1807	0.2748	0.063*	

### Atomic displacement parameters (Å^2^)

**Table:**

	*U*^11^	*U*^22^	*U*^33^	*U*^12^	*U*^13^	*U*^23^
Cu1	0.0319 (4)	0.0335 (4)	0.0395 (4)	0.0032 (2)	0.0092 (3)	0.0029 (3)
Cr1	0.0290 (4)	0.0312 (4)	0.0411 (5)	−0.0031 (3)	0.0101 (4)	0.0001 (3)
S1	0.0677 (13)	0.0636 (12)	0.177 (3)	0.0141 (10)	0.0743 (16)	0.0278 (14)
S2	0.0747 (14)	0.1043 (17)	0.1101 (17)	−0.0397 (12)	0.0634 (13)	−0.0378 (14)
N1	0.049 (3)	0.056 (3)	0.049 (3)	0.006 (2)	0.024 (2)	0.009 (2)
N2	0.041 (3)	0.052 (3)	0.071 (3)	−0.007 (2)	0.026 (3)	0.001 (3)
N3	0.044 (3)	0.045 (3)	0.035 (2)	0.009 (2)	0.001 (2)	0.003 (2)
N4	0.044 (3)	0.034 (2)	0.047 (3)	−0.009 (2)	0.010 (2)	−0.002 (2)
O1	0.0272 (18)	0.0287 (17)	0.0445 (19)	0.0019 (14)	0.0100 (15)	0.0030 (15)
O2	0.049 (2)	0.040 (2)	0.043 (2)	−0.0046 (18)	0.0085 (18)	0.0030 (16)
O3	0.0278 (19)	0.049 (2)	0.052 (2)	−0.0018 (16)	0.0070 (17)	0.0112 (18)
O4	0.042 (2)	0.0336 (19)	0.048 (2)	−0.0010 (16)	0.0045 (17)	0.0009 (16)
O5	0.066 (3)	0.062 (3)	0.079 (3)	−0.009 (2)	0.025 (3)	−0.015 (3)
C1	0.049 (4)	0.034 (3)	0.061 (4)	−0.001 (3)	0.019 (3)	0.005 (3)
C2	0.049 (4)	0.046 (3)	0.058 (4)	−0.003 (3)	0.026 (3)	−0.013 (3)
C3	0.039 (3)	0.032 (3)	0.066 (4)	0.006 (2)	0.021 (3)	0.002 (3)
C4	0.057 (4)	0.035 (3)	0.067 (4)	−0.005 (3)	0.029 (3)	−0.009 (3)
C5A	0.043 (12)	0.041 (13)	0.076 (13)	−0.023 (8)	−0.002 (11)	0.000 (9)
C6A	0.079 (12)	0.015 (7)	0.053 (9)	−0.008 (8)	0.020 (8)	0.011 (6)
C5B	0.076 (17)	0.049 (18)	0.11 (2)	−0.035 (13)	0.053 (17)	−0.049 (19)
C6B	0.13 (2)	0.039 (11)	0.079 (14)	−0.018 (13)	0.041 (14)	0.014 (9)
C7A	0.035 (9)	0.046 (9)	0.055 (9)	−0.007 (5)	−0.011 (9)	0.015 (6)
C7B	0.077 (17)	0.043 (9)	0.064 (11)	−0.008 (7)	−0.022 (14)	0.019 (8)
C8	0.020 (3)	0.060 (4)	0.067 (4)	−0.005 (2)	0.000 (3)	0.012 (3)
C9	0.040 (3)	0.057 (4)	0.058 (4)	0.006 (3)	−0.009 (3)	0.001 (3)
C10	0.079 (5)	0.079 (5)	0.036 (3)	0.026 (4)	0.004 (3)	0.006 (3)
C11	0.046 (3)	0.042 (3)	0.053 (3)	0.008 (3)	0.013 (3)	0.003 (3)
C12	0.057 (4)	0.033 (3)	0.067 (4)	0.000 (3)	0.014 (3)	0.007 (3)

### Geometric parameters (Å, º)

**Table:**

Cu1—O3	1.909 (4)	C3—H3A	0.9700
Cu1—N1	1.938 (5)	C3—H3B	0.9700
Cu1—O1	1.994 (3)	C4—H4A	0.9700
Cu1—N3	2.064 (4)	C4—H4B	0.9700
Cu1—O4	2.259 (4)	C5A—H5AA	0.9600
Cu1—Cr1	2.9979 (11)	C5A—H5AB	0.9600
Cr1—O2	1.912 (4)	C5A—H5AC	0.9600
Cr1—O3	1.961 (4)	C6A—C7A	1.507 (5)
Cr1—O1	1.984 (3)	C6A—H6AA	0.9700
Cr1—N2	2.012 (5)	C6A—H6AB	0.9700
Cr1—O5	2.071 (5)	C5B—H5BA	0.9600
Cr1—N4	2.118 (5)	C5B—H5BB	0.9600
S1—C1	1.610 (7)	C5B—H5BC	0.9600
S2—C2	1.636 (6)	C6B—C7B	1.507 (5)
N1—C1	1.150 (8)	C6B—H6BA	0.9700
N2—C2	1.124 (8)	C6B—H6BB	0.9700
N3—C10	1.481 (8)	C7A—H7AA	0.9700
N3—C9	1.481 (8)	C7A—H7AB	0.9700
N3—C11	1.484 (7)	C7B—H7BA	0.9700
N4—C4	1.469 (8)	C7B—H7BB	0.9700
N4—C6B	1.481 (5)	C8—C9	1.510 (9)
N4—C6A	1.483 (5)	C8—H8A	0.9700
N4—C5B	1.48 (4)	C8—H8B	0.9700
N4—C5A	1.51 (4)	C9—H9A	0.9700
O1—C3	1.431 (6)	C9—H9B	0.9700
O2—C7A	1.420 (5)	C10—H10A	0.9600
O2—C7B	1.420 (5)	C10—H10B	0.9600
O3—C8	1.419 (6)	C10—H10C	0.9600
O4—C12	1.430 (6)	C11—C12	1.504 (4)
O4—H4	0.8635	C11—H11A	0.9700
O5—H5A	0.8379	C11—H11B	0.9700
O5—H5B	0.8680	C12—H12A	0.9700
C3—C4	1.522 (8)	C12—H12B	0.9700
			
O3—Cu1—N1	159.1 (2)	C4—C3—H3B	110.0
O3—Cu1—O1	79.60 (14)	H3A—C3—H3B	108.3
N1—Cu1—O1	96.24 (18)	N4—C4—C3	110.6 (5)
O3—Cu1—N3	83.98 (18)	N4—C4—H4A	109.5
N1—Cu1—N3	100.4 (2)	C3—C4—H4A	109.5
O1—Cu1—N3	163.20 (17)	N4—C4—H4B	109.5
O3—Cu1—O4	105.57 (16)	C3—C4—H4B	109.5
N1—Cu1—O4	95.29 (19)	H4A—C4—H4B	108.1
O1—Cu1—O4	98.81 (14)	N4—C5A—H5AA	109.5
N3—Cu1—O4	82.15 (16)	N4—C5A—H5AB	109.5
O3—Cu1—Cr1	39.87 (11)	H5AA—C5A—H5AB	109.5
N1—Cu1—Cr1	136.55 (15)	N4—C5A—H5AC	109.5
O1—Cu1—Cr1	40.97 (10)	H5AA—C5A—H5AC	109.5
N3—Cu1—Cr1	122.24 (14)	H5AB—C5A—H5AC	109.5
O4—Cu1—Cr1	98.30 (10)	N4—C6A—C7A	108.3 (16)
O2—Cr1—O3	172.67 (17)	N4—C6A—H6AA	110.0
O2—Cr1—O1	94.96 (16)	C7A—C6A—H6AA	110.0
O3—Cr1—O1	78.60 (15)	N4—C6A—H6AB	110.0
O2—Cr1—N2	92.6 (2)	C7A—C6A—H6AB	110.0
O3—Cr1—N2	93.75 (19)	H6AA—C6A—H6AB	108.4
O1—Cr1—N2	172.14 (19)	N4—C5B—H5BA	109.5
O2—Cr1—O5	90.49 (18)	N4—C5B—H5BB	109.5
O3—Cr1—O5	93.16 (19)	H5BA—C5B—H5BB	109.5
O1—Cr1—O5	91.57 (17)	N4—C5B—H5BC	109.5
N2—Cr1—O5	90.6 (2)	H5BA—C5B—H5BC	109.5
O2—Cr1—N4	82.93 (16)	H5BB—C5B—H5BC	109.5
O3—Cr1—N4	92.86 (17)	N4—C6B—C7B	110.4 (18)
O1—Cr1—N4	84.21 (16)	N4—C6B—H6BA	109.6
N2—Cr1—N4	94.5 (2)	C7B—C6B—H6BA	109.6
O5—Cr1—N4	171.83 (19)	N4—C6B—H6BB	109.6
O2—Cr1—Cu1	135.64 (13)	C7B—C6B—H6BB	109.6
O3—Cr1—Cu1	38.60 (11)	H6BA—C6B—H6BB	108.1
O1—Cr1—Cu1	41.21 (10)	O2—C7A—C6A	106.3 (16)
N2—Cr1—Cu1	131.57 (16)	O2—C7A—H7AA	110.5
O5—Cr1—Cu1	85.52 (15)	C6A—C7A—H7AA	110.5
N4—Cr1—Cu1	95.78 (12)	O2—C7A—H7AB	110.5
C1—N1—Cu1	159.7 (5)	C6A—C7A—H7AB	110.5
C2—N2—Cr1	174.5 (5)	H7AA—C7A—H7AB	108.7
C10—N3—C9	110.3 (5)	O2—C7B—C6B	112.2 (19)
C10—N3—C11	109.4 (5)	O2—C7B—H7BA	109.2
C9—N3—C11	110.5 (5)	C6B—C7B—H7BA	109.2
C10—N3—Cu1	113.8 (4)	O2—C7B—H7BB	109.2
C9—N3—Cu1	104.6 (3)	C6B—C7B—H7BB	109.2
C11—N3—Cu1	108.1 (3)	H7BA—C7B—H7BB	107.9
C4—N4—C6B	122.3 (13)	O3—C8—C9	106.3 (5)
C4—N4—C6A	102.8 (9)	O3—C8—H8A	110.5
C4—N4—C5B	104.2 (17)	C9—C8—H8A	110.5
C6B—N4—C5B	108 (2)	O3—C8—H8B	110.5
C4—N4—C5A	113.2 (18)	C9—C8—H8B	110.5
C6A—N4—C5A	112.0 (18)	H8A—C8—H8B	108.7
C4—N4—Cr1	105.7 (3)	N3—C9—C8	109.8 (5)
C6B—N4—Cr1	105.4 (13)	N3—C9—H9A	109.7
C6A—N4—Cr1	106.1 (9)	C8—C9—H9A	109.7
C5B—N4—Cr1	110.9 (15)	N3—C9—H9B	109.7
C5A—N4—Cr1	115.9 (16)	C8—C9—H9B	109.7
C3—O1—Cr1	110.9 (3)	H9A—C9—H9B	108.2
C3—O1—Cu1	116.8 (3)	N3—C10—H10A	109.5
Cr1—O1—Cu1	97.82 (14)	N3—C10—H10B	109.5
C7A—O2—Cr1	108.6 (9)	H10A—C10—H10B	109.5
C7B—O2—Cr1	117.0 (10)	N3—C10—H10C	109.5
C8—O3—Cu1	115.7 (3)	H10A—C10—H10C	109.5
C8—O3—Cr1	136.0 (4)	H10B—C10—H10C	109.5
Cu1—O3—Cr1	101.53 (16)	N3—C11—C12	111.9 (5)
C12—O4—Cu1	103.0 (3)	N3—C11—H11A	109.2
C12—O4—H4	106.9	C12—C11—H11A	109.2
Cu1—O4—H4	140.0	N3—C11—H11B	109.2
Cr1—O5—H5A	111.1	C12—C11—H11B	109.2
Cr1—O5—H5B	109.4	H11A—C11—H11B	107.9
H5A—O5—H5B	110.2	O4—C12—C11	108.2 (4)
N1—C1—S1	177.1 (6)	O4—C12—H12A	110.1
N2—C2—S2	177.8 (6)	C11—C12—H12A	110.1
O1—C3—C4	108.6 (4)	O4—C12—H12B	110.1
O1—C3—H3A	110.0	C11—C12—H12B	110.1
C4—C3—H3A	110.0	H12A—C12—H12B	108.4
O1—C3—H3B	110.0		
			
Cr1—O1—C3—C4	−40.1 (5)	N4—C6A—C7A—O2	52.7 (17)
Cu1—O1—C3—C4	70.8 (5)	Cr1—O2—C7B—C6B	11 (2)
C6B—N4—C4—C3	86.1 (13)	N4—C6B—C7B—O2	−33 (3)
C6A—N4—C4—C3	76.9 (10)	Cu1—O3—C8—C9	−32.5 (6)
C5B—N4—C4—C3	−151.0 (17)	Cr1—O3—C8—C9	−177.5 (4)
C5A—N4—C4—C3	−162.0 (17)	C10—N3—C9—C8	−163.4 (5)
Cr1—N4—C4—C3	−34.1 (5)	C11—N3—C9—C8	75.5 (6)
O1—C3—C4—N4	50.2 (6)	Cu1—N3—C9—C8	−40.6 (6)
C4—N4—C6A—C7A	−136.0 (11)	O3—C8—C9—N3	48.3 (7)
C5A—N4—C6A—C7A	102 (2)	C10—N3—C11—C12	88.3 (6)
Cr1—N4—C6A—C7A	−25.3 (14)	C9—N3—C11—C12	−150.1 (5)
C4—N4—C6B—C7B	−84 (2)	Cu1—N3—C11—C12	−36.2 (6)
C5B—N4—C6B—C7B	155 (2)	Cu1—O4—C12—C11	−46.7 (5)
Cr1—N4—C6B—C7B	36 (2)	N3—C11—C12—O4	59.0 (7)
Cr1—O2—C7A—C6A	−55.6 (12)		

### Hydrogen-bond geometry (Å, º)

**Table:**

*D*—H···*A*	*D*—H	H···*A*	*D*···*A*	*D*—H···*A*
O4—H4···O2^i^	0.86	1.86	2.595 (7)	142
O5—H5*B*···O1^i^	0.86	2.18	3.014 (7)	162

Symmetry code: (i) −*x*, −*y*, −*z*.
